# Alpha linolenic acid in maternal diet halts the lipid disarray due to saturated fatty acids in the liver of mice offspring at weaning

**DOI:** 10.1186/s12944-015-0012-7

**Published:** 2015-02-26

**Authors:** Limor Shomonov-Wagner, Amiram Raz, Alicia Leikin-Frenkel

**Affiliations:** Laboratory for Lipid Metabolism in the Liver, Sackler School of Medicine, Tel Aviv, 69978 Israel; G.S.W. Faculty of Life Sciences, Tel Aviv University, Tel Aviv, 69978 Israel; Bert W. Strassburger Lipid Center, Sheba Medical Center, Tel-Hashomer, Israel

**Keywords:** Maternal diet, Omega (ω) -3 fatty acids, Saturated fatty acids, Nutrition, Liver, Lipids, Fatty acids/desaturases, Programming, Insulin resistance

## Abstract

**Background:**

Alpha linolenic acid (ALA, 18:3) in maternal diets has been shown to attenuate obesity associated insulin resistance (IR) in adult offspring in mice. The objective in the present study was to detect the early effects of maternal dietary saturated fatty acids (SFA) and their partial substitution with ω-3 ALA, docosa hexenoic acid (DHA,22:6) and eicosapentenoic acid 20:5 (EPA,20:5) on the HOMA index, liver lipids and fatty acid desaturases in the offspring at weaning.

**Methods:**

3 month old C57Bl6/J female mice were fed diets containing normal amount of calories but rich in SFA alone or partially replaced with ALA, DHA or EPA before mating, during pregnancy and lactation.

**Results:**

Pregnant mice fed SFA produced offspring with the highest HOMA index, liver lipids and desaturase activities. ALA prevented SFA induced lipid increase but DHA and EPA only reduced it by 42% and 31% respectively. ALA, DHA and EPA decreased HOMA index by 84%, 75% and 83% respectively. ALA, DHA and EPA decreased Δ6 and SCD1 desaturase activities about 30%.

**Conclusions:**

SFA feeding to mothers predisposes their offspring to develop IR and liver lipid accumulation already at weaning. ω3 fatty acids reduce IR, ALA halts lipid accumulation whereas DHA and EPA only blunt it.ALA and DHA restore the increased SCD1 to normal. These studies suggest that ω-3 fatty acids have different potencies to preclude lipid accumulation in the offspring partially by affecting pathways associated to SCD1 modulation.

## Background

Metabolic syndrome and cardiometabolic risk have progressively become a major public health problem [1]. Lifestyles factors such as diet, increasing maternal age, endocrine disruption, etc. were suggested [2] to affect physiological and metabolic processes and the underlying genetic networks in tissue specific manner [3,4]. Increased biosynthesis and reduced oxidation of FAs triggers obesity development and obesity-related complications including insulin resistance (IR), and the metabolic syndrome [5,6]. High consumption of dietary fats also contributes to these diseases. However, recent evidence indicates that the quality, beyond the quantity, of dietary FAs affects differentially some of these metabolic parameters with SFA having shown to increase lipogenesis and the expression of responsible genes in the liver and adipose tissue [7,8]. In contrast there is evidence to suggest that essential FA i.e., ALA and linoleic acid ( LA:18:2 ω-6) [9] and the PUFA derivatives, DHA and EPA reduce obesity- associated risk by promoting an increase in FA oxidation [10,11].

The impact of dietary fatty acids on developmental origins of metabolic disease is still poorly understood. The Fetal Origins hypothesis states that several of the major diseases of adult life originate in impaired intra-uterine growth and development [12-14]. Accumulating evidence in both human and animal model indicates that ω-3 fatty acids EPA and DHA do have distinct capabilities to modulate both cellular metabolic functions and gene expression [15]. Thus Δ6 desaturase, key enzyme in the conversion of ALA to EPA and DHA is significantly reduced in obesity, IR, and metabolic syndrome [16,17]. On the other hand SCD1 that converts saturated into monounsaturated fatty acids is increased in obesity, NAFLD and IR [18–20]. During mammalian development, FAs consumed by the mother are transferred to the fetus through the placenta. Therefore, FAs present in the fetus reflect the maternal diet and also it’s own metabolic products [21-25]. Thus, the quality and composition of dietary FAs, in addition to the quantity consumed, may have critical roles on the regulation of metabolic and/or endocrine pathways during fetal development. This is particularly relevant for the essential ALA and LA, which cannot be synthesized in mammals and must be therefore obligatory consumed in the diet [9]. Several studies have shown that supplementing the diet of mothers with ω-3 PUFA during pregnancy led to a reduction in fat mass in the offspring [26,27] and decreased inflammation. We have recently reported [28] that enriching maternal diet of mice during pregnancy-lactation with ALA prevented obesity-associated insulin resistance and fatty liver in the adult offspring when challenged with a high fat diet (HFD).In contrast, providing mothers an SFA-enriched diet exasperated the metabolic dysfunctions caused by a HFD in adult offspring. In light of these findings, the effect of exposure to different fatty acids classes’ quality, rather than quantity, during the perinatal period on lipid metabolism in the offspring calls for further investigation.

The hypothesis of the current study is that maternal consumption during pregnancy of diets enriched with ALA and, possibly, other ω-3 fatty acids may prevent the early fetal development of SFA-induced lipid derangements leading to long term disease in the offspring. We question here the differential involvement of maternal dietary ω-3 ALA, DHA or EPA in the prevention of IR and hepatic lipid accumulation and the contribution of fatty acid desaturases in the process in weaning offspring. We show that ALA is the most efficient ω-3 fatty acid in the prevention of liver lipids accumulation and that the normalization of desaturases may contribute to the involved mechanisms.

## Results

This study examined the effects of feeding five groups of mothers diets containing 6 g% fat and the same amount of calories equivalent to those in a regular chow diet but differing only in the fatty acid composition confirmed by GC as described in Methods.

Dams health was not affected by the maternal diets and the same number of pups were fed per mother during lactation for all dietary treatments.

### Maternal dietary fatty acid composition affected differently glucose and insulin in the offspring

**Figure 1:**
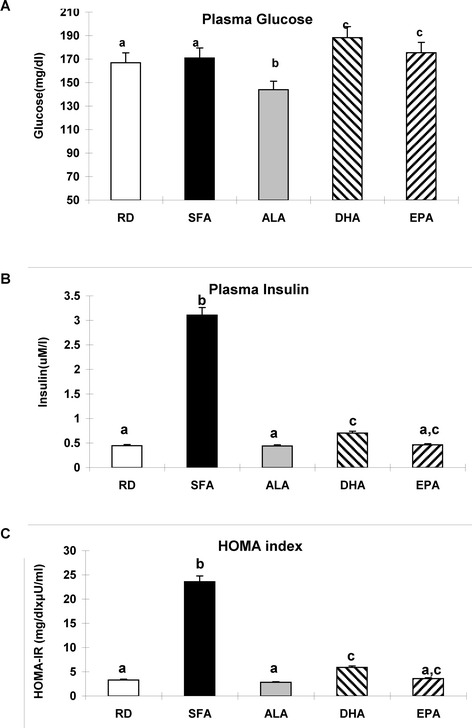
**Insulin resistance parameters in the offspring. A** - Plasma Glucose levels, **B**- Plasma Insulin levels and **C**-HOMA index in offspring at day 21, after weaning. Means (n = 7) without a common letter difference p < 0.05 between the maternal dietary treatments indicated in the x-axis, as described in Methods.

Plasma glucose was slightly but significantly lower in ALA compared to the other diets. Significantly, plasma insulin and HOMA index were almost 6 fold higher in SFA group than in RD and ALA (Figure 1A,B,C). DHA and EPA also lowered glucose and HOMA index.

### Maternal dietary fatty acid composition differentially affected liver weight in the offspring

**Figure 2:**
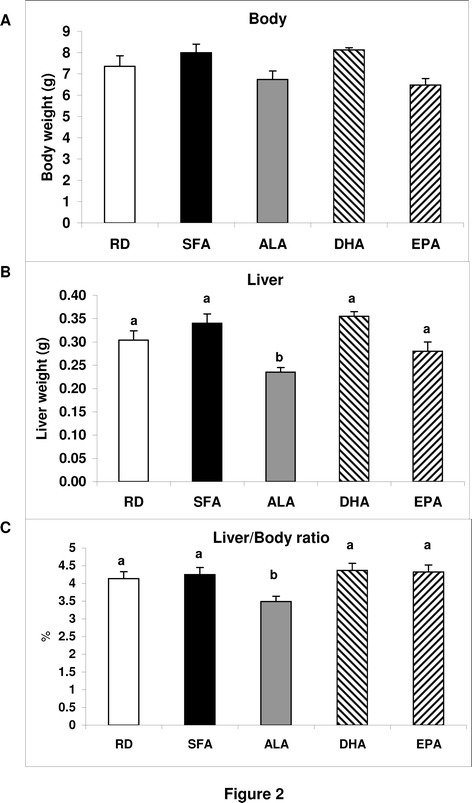
**Offspring body weigh, liver weight and liver/body ratio.** The offspring body weight **(A)**, liver weight **(B)** and their ratio **(C)** at weaning are shown as means (n = 8 RD,12SFA,21ALA,7 DHA and 15 EPA). Those without a common letter differ p < 0.05 between the maternal dietary treatments indicated in the x-axis, as described in Methods.

In line with the above data, total liver weight and liver/body ratio of the offspring at weaning were about 15% and 25%, respectively, lower for ALA animals than for the other dietary treatments (Figure 2B and C), while the animals were otherwise healthy.

### Maternal dietary fatty acids differentially affected liver lipid content and lipid classes’ distribution

**Figure 3:**
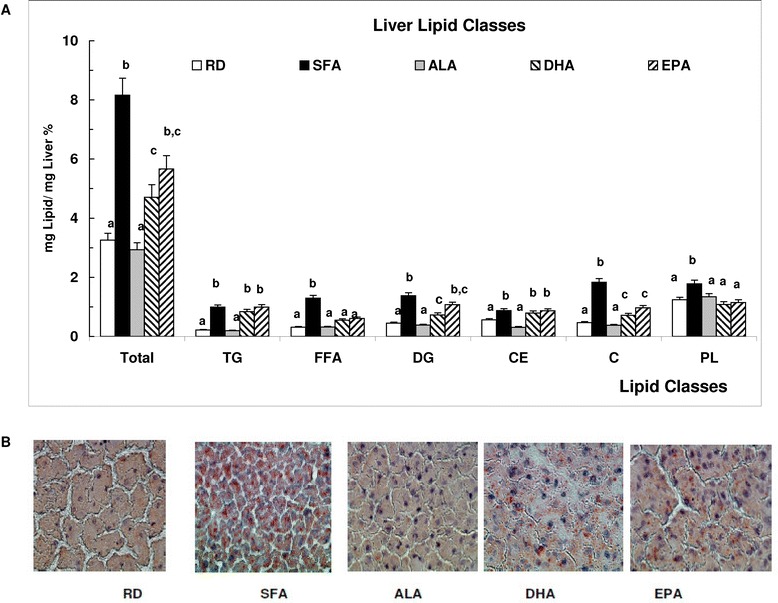
**Liver lipids. A** – Liver lipid classes’ distribution expressed as percent lipids of the total liver mass in the offspring offspring at day 21, after weaning. Means (n = 7) without a common letter difference p < 0.05. **B**- Liver neutral lipids staining with ORO and observed by light microscopy with 60× magnification.

The lower liver/body ratio in ALA offspring suggested the possibility of lower lipid content in the liver of those animals. Indeed, TLL were three times higher in SFA than in RD and ALA and indicative of steatosis DHA and EPA had 50% and 30% lower liver fat, respectively, than SFA but still significantly higher than ALA and RD (Figure 3A). The lower amount of total fat was due mainly to a decrease in TG seen by TLC (Figure 3A) and confirmed by Oil Red O staining (Figure 3B). Other lipids classes, among them free fatty acids, diacylglycerides and free cholesterol, were significantly higher in SFA than in other treatments while they were similar in RD and ALA. TLL higher than 5%, was developed in animals fed SFA, DHA and EPA while prevented by ALA.

**Figure 4:**
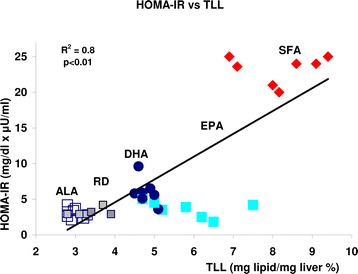
**Correlation between HOMA-IR and TLL in the offspring of mothers fed different diets, represented by different symbols/Empty squares: ALA, framed filled squares RD, filled circles: DGA, filled large squares:EPA, diamonds: SFA.**

Interestingly, Figure 4 shows a significant correlation between TLL and HOMA index. It can be seen clearly here that in ALA and RD both parameters were lower than in other diets.SFA had the higher parameters, sitting high in the correlation graph.

### Maternal dietary fatty acids differentially modified liver fatty acid composition and fatty acid desaturases in the offspring

TLL fatty acid analysis showed that in the SFA offspring there were higher (n.s.) levels of SFAs and MUFA and lower levels of PUFA, compared with the other diets (Table 1) except for, surprisingly, EPA. In turn DHA offspring had the highest (n.s) amount of total PUFAs.

**Table 1:** **Offspring liver fatty acid composition**

			**Fatty acid abundance (mol %)**			
**Fatty acids in liver lipids**		**RD**	**SFA**	**ALA**	**DHA**	**EPA**
Saturated fatty acids						
14:00	Myrisitic acid	1.0 ± 0.1	0.8 ± 0.07	0.8 ± 0.08	0.6 ± 0.07	0.7 ± 0.06
16:00	Palmitic acid	21.4 ± 1.9	19.6 ± 1.8	20.6 ± 1.8	19.4 ± 1.6	19.9 ± 2.0
18:00	Stearic acid	17.7 ± 1.1	22.4 ± 2.0	19.4 ± 2.1	18.6 ± 1.8	21.6 ± 1.8
**Total SFAs**		40.2 ± 2.9	42.8 ± 3.9	40.9 ± 2.5	38.7 ± 3.4	42.3 ± 3.9
Monounsaturated fatty acids (MUFA)						
16:1ω-7	Palmitoleic acid	0.4 ± 0.05	0.7 ± 0.08	0.5 ± 0.06	0.5 ± 0.06	0.7 ± 0.08
18:1 ω -9	Oleic acid	9.2 ± 0.9	10.1 ± 1.1	7.1 ± 0.8	6.3 ± 0.5	7.6 ± 0.8
**Total MUFA**		9.6 ± 0.9	10.9 ± 0.9	7.7 ± 0.7	6.8 ± 0.5	8.3 ± 0.7
Polyunsaturated fatty acids (PUFA)						
18:2 ω6	Linoleic	27.4 ± 1.9^a^	15.4 ± 1.4^b^	17.0 ± 1.5^b^	13.6 ± 1.4^b^	14.8 ± 1.5^b^
18:3 ω6	γ- Linolenic acid	0.4 ± 0.05^a^	0.1 ± 0.01^b^	0.1 ± 0.01^b^	0.0 ± 0.0	0.0 ± 0.0
20:4 ω6	Arachidonic acid	11.3 ± 1.3^a^	16.5 ± 1.5^b^	12.2 ± 1.1^a^	9.5 ± 0.8^c^	8.2 ± 0.8^c^
**Total n-6 PUFA**		39.2 ± 3.9^a^	32.2 ± 3.2^a^	29.4 ± 2.9^a,b^	23.3 ± 2.3^b^	23.0 ± 2.2^b^
18:3 ω3	α-Linolenic acid	1.1 ± 0.1^a^	0.1 ± 0.01^b^	1.7 ± 0.2^a^	0.1 ± 0.01^b^	0.2 ± 0.02^b^
20:5 ω3	Eicosapentenoic acid	0.4 ± 0.03	0.4 ± 0.04	3.1 ± 0.03	2.1 ± 0.2	7.1 ± 0.5
22:6 ω3	Docosahexenoic acid	9.3 ± 0.9	13.3 ± 1.2	16.9 ± 1.7	28.7 ± 2.5	18.7 ± 1.9
Total ω n-3 PUFA		10.9 ± 1.1^a^	13.9 ± 1.2^a^	21.9 ± 2.2^b^	31.0 ± 3.1^c^	26.1 ± 2.5^c^
**Total PUFA**		50.1 ± 4.9	46.2 ± 4.6	51.3 ± 4.8	54.3 ± 4.9	49.2 ± ±4.7

Fatty acids are expressed as mole %. Fatty acids changed among the different maternal dietary treatments appear in bold. Means (n = 7) without a common letter (a, b or c) difference p < 0.05 between the maternal dietary treatments indicated in the x-axis, as described in Methods.

**Figure 5:**
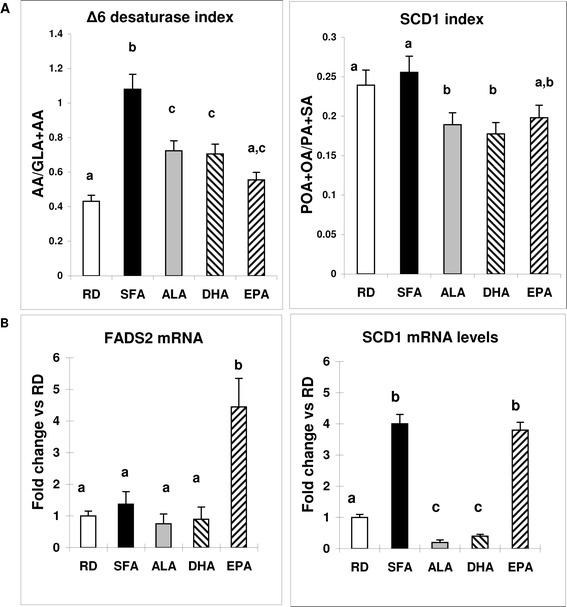
**Δ6 desaturase and SCD1 index.** FADS2 and SCD1 mRNA levels. **A** - Represents the liver Δ6 desaturase index calculated from the plasma fatty acids profile for linoleic acid (18:2) and it’s products of desaturation γ-linolenic (18:3) and desaturation/elongation arachidonic (20:4 ) acids (left) and the SCD1 index calculated from the plasma fatty acids profile for palmitic (16:0) and stearic acids (18:0) and their monounsaturated products, palmitoleic (16:1) and oleic (18:1) (right). **B** - Represents the FADS2 (left) and SCD1 mRNA (right) expression levels offspring at day 21, after weaning in liver, calculated as fold change compared to the RD in the same group. Means (n = 7) without a common letter difference p < 0.05. The x-axis indicates that the different maternal diets received by animals, as described in Methods and the determinations were normalized by actin levels and calculated as fold change compared to the RD in the same group.

Fatty acid desaturases are mostly active in the liver and their activities can be calculated from the surrogate markers in the fatty acid profile. Δ6 desaturase index, calculated from the surrogate markers [GLA + AA/LA] was significantly higher in SFA than in other groups (Figure 5A left). SCD1 index, calculated from the surrogate markers in the plasma fatty acids profile [POA + OA/PA + SA] was decreased by the ω-3 FA diets (Figure 5A right). Similar to our previous work [28] and with comparative purposes, liver FADS2 mRNA expression levels were calculated as fold change in different maternal dietary treatments related to those in RD diet after normalization to actin. ALA and DHA had significantly lower values than other groups whereas EPA, unexpectedly, had significant higher values than all other diets (Figure 5B left). Liver SCD1 mRNA expression levels in SFA and EPA were significantly higher than other diets (Figure 5B right).

## Discussion

In this paper the role of fatty acids in maternal diets and their effects on the liver lipid metabolism and health status of the offspring was investigated by biochemical and metabolic approaches. We found that SFA in maternal diets predisposed the 21 days weaning offspring to IR and liver lipids accumulation whereas partial replacement with ALA halted those changes. Interestingly, DHA and EPA, while preventing IR, only partially reduced lipid accumulation.

One of the most striking features of the phenotype in the model used is that maternal SFA induced lipid accumulation in the weaning offspring in the liver in the absence of changes in body weight. This may point to the liver as the first sensitive organ during development to sense the SFA detrimental effects [3]. Moreover, this study shows the distinct beneficial effects of partial replacement of SFA with ALA, DHA or EPA on the modulation of the induced lipid disarray. Similar to our previous study [28] and different from other animal models previously described [29] the dams in our study were healthy during pregnancy. Therefore, the effects of the maternal diets on the offspring can be directly associated with the differential fatty acids’ composition of the maternal diets. HOMA index, a direct indicator of insulin resistance, was increased in the SFA offspring but not in the ω3 FAs offspring. These effects were also previously observed in the adult offspring but only after a HFD induced obesity [28]. They are also in line with the observed detrimental effects of dietary SFA on IR when provided directly to adult animals as previously described by others [30]. Since in this study the offsprings were not obese at weaning, the present data strongly imply that SFA in maternal diets had a direct per se effect on the embryional physiological and metabolic pathways involved in programming insulin resistance in the offspring [31].

Our results show that SFA in maternal diet predispose liver lipid increase in the weaning offspring, independent of body weight modifications (Figure 3A,B). An accumulation of saturated fatty acids in the liver may play an important role in the beginning of steatosis and a growing body of literature suggests that saturated fatty acids may initiate the toxic effects leading to the pathogenesis of NAFLD, impaired liver function and ultimately liver failure [32,33]. The lower liver weight and liver/body ratio in otherwise healthy ALA animals (Figure 2B and C) was linked to the lack of additional fat in the liver of ALA offspring compared to RD and 100% significantly lower TLL than SFA offspring. DHA and EPA, in turn, had lower amount than SFA but still, higher than RD, (Figure 3A,B). Hepato-steatosis may lead to primary NAFLD that, in turn, may be associated with IR and may also precede NAFLD and its phenotypic manifestations [34]. In the present study both conditions were found to be induced by maternal dietary SFA in the offspring and decreased by the partial replacement of SFA with ALA (Figures 3 and 4). The significant correlation observed between HOMA-IR and TLL (Figure 4) may suggest that ALA interferes with the mechanism/s by which SFA induce lipid increment in the offspring [35] differently than DHA and EPA.

Recent reviews on the effects of ω-3 PUFA provided directly to adult animals, on metabolic diseases, have focused primarily on the effects of EPA and DHA [36,37]. Furthermore, studies on the beneficial effects of ALA in the context of NAFLD suggest that these effects are solely due to the conversion of ALA to the long-chain products EPA and DHA. Our results, however, support a direct beneficiary effect by dietary ALA, independent of its longer chain metabolic products ( the conversion of ALA to DHA and EPA was 68% and 12.5% respectively). These results are in agreement of others in which ALA was fed to adult animals [38,39]. Possible mechanisms for the beneficiary effects of ALA on preventing liver lipid accumulation vs. the detrimental effects of dietary SFA may be due, at least in part, to their differential abilities to modulate metabolic pathways leading to higher lipid synthesis/accumulation [40] or to promote FA oxidation [10,11]. However, possible mechanisms involving PPARα, and the subsequent regulatory pathways, have already been shown in adult mice not to account for the ALA beneficial effect [41].

The liver fatty acid profiles measured in this work would indicate that the maternal dietary fatty acids were indeed transferred to the offspring and distinctly metabolized. Maternal ω-3PUFA led to a decrease of total MUFA and ω-6PUFA with a significant increase in total ω-3FA compared to RD and SFA in liver offspring. Moreover, not only ALA was the highest in the ALA fed offspring but it was also significantly converted into EPA and DHA. DHA was shown to be the highest in the DHA fed offspring and represented most of the ω-3PUFA. A very low amount of EPA was formed probably by retro conversion [42]. EPA, in turn, was the highest in the EPA fed offspring and was also significantly converted into DHA. However, neither DHA nor EPA as dietary components, achieved the potency shown by ALA to prevent lipid increase, thus highlighting the distinctiveness of each of the ω-3FA tested, as also addressed by others [43].

Maternal SFA induced an increase in Δ6 desaturase products γ-linolenic and arachidonic acids (implying also Δ5 desaturase) in the offspring. This increase was significantly blunted by ω-3FAs. Maternal SFA induced an increase in SCD1 monounsaturated 16:1 and 18:1 products, compared to RD, that was significantly decreased by ω-3FA maternal diets. The gene expression levels of FADS2 were lower in the ALA and DHA fed animals than in SFA fed ones, whereas in the EPA-fed group they were unexpectedly higher. SCD1 mRNA levels were significantly increased by SFA compared to RD and significantly reduced for ALA and DHA but, surprisingly, unchanged for EPA. SCD1 deficiency has been shown to activate metabolic pathways that promote β-oxidation and decrease lipogenesis in liver and skeletal muscles and possibly lower IR [44]. In turn, a decrease in Δ6 desaturase activity, has been found to be correlated with reduced inflammatory processes [45]. The observed lack of correlation found in this work between the activity and mRNA expression for FAD2 and SCD1 in the EPA-fed group may involve a post-translational modification or inhibitory conditions for the enzyme activities. Our results hint to the potential of dietary fatty acids ALA, DHA and EPA, in pregnancy, to differentially affect FADS2 and SCD1 expression, activity and also pathways that lower lipid accumulation [20,22]. We have found the active involvement of the desaturases in the offspring processing of the ω-3FA consumed in the maternal diets. However, the similarities of their response do not support completely their different ability to prevent (ALA) or reduce (DHA and EPA) liver lipid levels. This apparent contradiction may point at different regulatory pathways [46-48] activated early by each of the maternal fatty acids in the foetuses or, maybe, to genetic variations [49,50]. Similarly, possible epigenetic modifications like those described for SCD1 [51] may be differentially triggered by maternal fatty acids and will be analysed in the future.

Even though this is still a relatively unexplored area, two conclusions emerge clearly: first, that maternal saturated fatty acids, independent of fat amount or calories, induce differential metabolic effects leading to liver lipid accumulation that can be detected in the offspring phenotype as early as 21 days of life; and second, that not only alpha linolenic acid and saturated fatty acids have divergent effects on insulin resistance and liver lipid levels but, moreover, different ω-3 fatty acids behave differently and should be referred to and studied individually and not as a group. Moreover, the alpha linolenic acid preventive effects observed in the present study appear to be, presently, unrelated to it’s conversion to docosa hexenoic acid and eicosa pentenoic acid. Stearoyl CoA desaturase1 decrease in alpha linolenic acid born offspring may account for a possible mechanism of reduction of lipogenesis. However, docosa hexenoic acid also decreases Stearoyl CoA desaturase1 but partially reduces total liver lipids. Other mechanisms should then be searched to explain the differences. They may be found associated to the different interaction between the tested fatty acids and nuclear factors involved in regulating the balance between fat storage and oxidation, namely LXRs, PPARs and target genes [52]. Although the molecular mechanism by which alpha linolenic acid prevents lipid accumulation in the liver has not been elucidated in this work, preliminary results from our laboratory may indicate that it up regulates the expression of genes involved in lipid oxidation.

Our present findings may have important clinical implications due to the vast number of biological pathways regulated by nutritional fatty acids [53-55]. These results shed light on the early time for detection and potential ways for the prevention of lipid-related metabolic disease in the offspring by means of addressing the maternal diet and its fatty acid composition.

## Materials and methods

### Animals and diets

C57Bl6/J mice, 4–5 weeks old, were obtained from the animal facility of Tel-Aviv University. The studies were approved by the Institutional Committee for Animal Experiments at Tel Aviv University, Tel Aviv, and Israel.

Female mice were fed five isocaloric diets.The control diet 1-RD contained 6 g% Soybean of commercial origin. The test diets contained 2-SFA 6 g% coconut oil alone: or a mixture of 4.2 g% coconut oil with 1.8 g% of ω3 fatty acids 3-ALA, 4-DHA and 5-EPA respectively. 99% pure fatty acids were a generous gift of Prof. Amiram Raz (Tel Aviv University). The composition of the various diets is presented in Table 2A. The dietary fatty acid (Table 2B) analysis showed that the proportion of SFA diet was 34.9% in SFA. ALA was 54.04.9% in ALA diet, DHA was 48.2% in DHA diet and EPA was 59.43% in EPA diet. The total ω-3/ω-6 ratio ranged from 0.1 in RD to 3.2 in the ALA diet.

**Table 2:** **Maternal diet composition**

**A**		**Dietary composition (g%)**					
		**Maternal diet**					
Protein		20.1					
Carbohydrate		53.8					
Fat *		6					
Other‡		20.1					
Total		100					
Calories from Fat (%)		12.6					
**B**			**Fatty acid abundance (mol%)**				
**Maternal diets**			**RD**	**SFA**	**ALA**	**DHA**	**EPA**
Saturated fatty acids							
14:00	Myrsitic acid		0.18	8.11	0.1	0.27	0
16:00	Palmitic acid		11.78	14.8	4.17	6.86	5.49
18:00	Stearic acid		4.1	12.03	7.7	2.3	1.82
**Total SFAs**			16.07	**34.93**	11.97	9.42	7.31
Monounsaturated fatty acids (MUFA)							
16:1ω-7	Palmitoleic acid		0.22	0.25	0.1	0.3	0.13
18:1ω-9	Oleic acid		24.97	18.34	16.52	13.45	10.53
**Total MUFA**			25.19	18.59	16.62	13.75	10.66
Polyunsaturated fatty acids (PUFA)							
18:2ω-6	Linoleic		52.09	41.06	16.64	25.74	20.21
18:3ω-6	Gamma linolenic acid		0.29	0.22	0.26	0	0
20:4ω-6	Arachidonic acid		0.47	0.36	0.15	0.31	0.21
**Total ω-6 PUFA**			**52.85**	41.64	17.05	26.05	20.42
18:3ω-3	Alpha linolenic acid		5.42	3.91	**54.04**	2.25	1.84
20:5ω-3	Eicosapentenoic acid		0.22	0.39	0.14	0.32	**59.43**
22:6ω-3	Docosahexenoic acid		0.25	0.54	0.19	**48.2**	0.33
**Total ω-3 PUFA**			5.89	4.84	54.36	50.78	61.61
**Total PUFA**			58.74	46.48	71.41	76.82	82.03
**ω3/ω6**			**0.11**	**0.12**	**3.19**	**1.95**	**3.02**

*Fat in maternal diet was Soybean Oil for RD, Coconut oil for SFA and Coconut oil (4.8%) and ALA, DHA or EPA (1.8%) for the diets under those names.

‡Fat free chow diet contained fiber, ash, aminoacides, minerals and vitamins added.

A - All diets fed to dams during pre-conception, pregnancy and lactation were prepared by mixing 94 g% fat-free chow diet with 6 g% different fats/oils: RD-soybean, SFA-coconut oil, ALA-ALA + coconut, DHA-DHA + coconut, EPA-EPA + coconut. Fat-free chow diet contained fiber, ash, aminoacides, minerals and vitamins.

B - Dietary Fatty acid composition: Fatty acid composition of the five diets provided to the dams in the present model RD, SFA, ALA, DHA and EPA provided to the mothers before and during pregnancy and lactation. The lower row presents the ratio between ω-3 and ω -6 fatty acids in each diet. The ω -3/ ω -6 ratio in each diet is indicated in bold.

C57Bl6/J females received the experimental diet two weeks pre-conception and during pregnancy and lactation. Males received regular chow diet except during the mating period during which they shared their female mates’ diets (1 male for every 2 females). The number of pups born per mother for different diets was 7 ± 1 for RD, SFA, ALA and 6 ± 1 for DHA and EPA. Mating was performed more than once. Pups were exposed to their mother’s diet during lactation. At day 21 (weaning) pups were euthanized by an overdose anesthetic (Xylazine-Ketanal), after an overnight (12 h) fasting. Liver samples were obtained under liquid nitrogen and were kept frozen (-70°C) until further use. Plasma was separated from the blood and kept frozen (-20°C) until use. At all stages, the animals had free access to food and water and were kept in ventilated rooms under a light/dark cycle of 12 h/12 h. Guidelines for the use and care of the animals at Tel Aviv University’s Animal House were followed. Food consumption and body weight were monitored weekly.

### Biochemical determinations

Plasma glucose levels were measured by using a Precision QID sensor MediSense (Abbott Laboratories Co MediSense Inc, Bedford, MA). Plasma insulin levels were determined after overnight fast (12 h) using an insulin immunoassay kit (MRC Mouse Insulin, Elisa 96 T, Mercodia AB, Sweden). The HOMA index was calculated as Plasma glucose [mg/dl] X Plasma Insulin [μU/ml]/22.5.

### Lipid extraction

Liver samples were weighed and homogenized with saline at a ratio of 1:5 (w: v) in plastic tubes on ice. Lipids were extracted from an aliquot of the liver homogenate according to the procedure of Folch et al. [56]. The total amount (total liver lipids, TLL) was calculated after aliquot evaporation to constant weight (18). TLL distribution was analyzed by TLC applying 50 μg aliquots. Samples were reconstituted in 50 μL of chloroform and spotted on Silica-G thin-layer chromatography (TLC) plates. Standards for each fraction were purchased from Sigma Aldrich (Rehovot, Israel ) and were spotted in separate TLC lanes (i.e., 25 μg of PL, TG, DG, CE and FFA). Plates were then placed in a 20x20 cm TLC chamber containing petroleum ether, ethyl ether, and acetic acid (80:20:1, v/v/v) and run for 45 min (18). PL, TG, DG, CE and FFA bands were visualized with Iodine, scanned and quantified (Epson V700). Neutral lipids were also detected in liver slices with the lysochrome ORO (C26H24N4O) and visualized by light microscopy with an x60 magnification [57].

### Fatty acid analysis

Fatty acids were analyzed as methyl ester derivatives (FAME) by gas chromatography (GC) in a Varian, 3800 Series (Walnut Creek, CA) chromatograph (FID) with a fused silica SGE capillary column 30 × 0.025 and Varian Star Workstation Advance Application software, version 6×. Aliquots of food lipids were processed like the liver for the analysis of fatty acids as follows: after lipid extraction and weight, aliquots representing 0.5 g food or 0.25 g of liver, kept frozen at -20°C before use, were taken into a screw-capped tube (teflon-lined) containing 5 μg heptadecanoic acid as Internal Standard. 1 ml 5% H2SO4 in methanol was added. The tubes were gassed with nitrogen, closed tightly and heated at 85°C for 1.5 h with occasional shaking. After cooling, 1 ml of hexane was added, the tubes’ content was mixed and, after a short centrifugation, the hexane layer was transferred into a new tube. Before GLC analysis, the hexane extracts were concentrated by evaporation under nitrogen. One-twentieth of the final re-suspension was applied in 1 μl hexane into the gas chromatograph. The fatty acid profiles were compared to that of a known mixture of fatty acids of animal source, PUFA2 (Supelco, USA) for identification (28).

### Δ6 desaturase and SCD1 activities

Δ6 desaturase and SCD1 activities were estimated as the indexes [58] calculated by determining the ratio between the final products of the pathway and substrates of the surrogate markers [GLA + AA/LA] and [POA + OA/PA + SA] respectively, taken from the liver.

### Δ6 desaturase and SCD1 mRNA expression

Total RNA was extracted using TriReagent (Sigma-Aldrich, Jerusalem, Israel) according to the manufacturer’s protocol. Extracted RNA underwent reverse transcription (RT) to form cDNA by means of the Verso™ RT-PCR Systems. The TaqMan® Gene Expression Assay-Pre-Made for FADS2, SCD-1 as well as Beta Actin were obtained from Applied Biosystems, Israel (Agentek Ltd). Reaction products were normalized according to expression of the Beta Actin gene and presented as fold change compared to the RD data in each group [28].

### Statistics analysis

For comparing the means of treatment amongst the maternal diet groups, one-way ANOVA analysis was implemented followed by an appropriate post hoc test: Tukey, using SSPS version 18. The number of animals used was RD =8, SFA = 12, ALA = 21, DHA = 7 and EPA = 15 for body and liver weight.The number of animals was 7 and representative of different mothers for all other determinations.Statistically significant results were reported according to a p value equal to or less than 0.05. All data are shown ± SEM unless stated otherwise. For relating HOMA-IR data with TLL data, a 2 tailed Pearson correlation analysis was performed.

## Abbreviations

Δ6 desaturase: Delta 6 desaturase enzyme
FADS2: Delta 6 desaturase gene
SCD1: Stearoyl-CoA desaturase enzyme and gene 1
FAs: Fatty acids
SFA: Saturated fatty acid
MUFA: Monounsaturated fatty acid
TG: Triglycerides
DG: Diglycerides
CE: Cholesterol ester
FFA: Free fatty acid
PA: Palmitic acid
POA: Palmitoleic acid
SA: Stearic acid
OA: Oleic acid
GLA: Gama linolenic acid
AA: Arachidonic acid
LA: Linoleic acid
GC: Gas-chromatography
NAFLD: Non alcoholic fatty liver disease
HFD: High fat diet
HOMA: Homeostatic model assessment
n.s: Not significant
PUFA: Polyunsaturated fatty acid
PL: Phospholipid
TLC: Thin layer chromatography, TLL, Total liver lipids
